# Programmed inappropriate ICD ventricular defibrillation for cardioversion of persistent atrial fibrillation

**DOI:** 10.1186/1757-1626-1-152

**Published:** 2008-09-12

**Authors:** Panagiotis Korantzopoulos, George Grekas, Thomas Pappas, John A Goudevenos

**Affiliations:** 1 Department of Cardiology, University of Ioannina School of Medicine, 45110 Ioannina, Greece

## Abstract

In this report we briefly describe a patient with a dual chamber implantable cardioverter defibrillator in the context of severe ischemic cardiomyopathy who developed persistent atrial fibrillation. After appropriate anticoagulation and under mild sedation the patient was successfully cardioverted to sinus rhythm after a programmed ventricular synchronized defibrillation using his defibrillator. Programmed internal cardioversion of persistent atrial fibrillation in patients who have an implantable cardioverter defibillator without atrial defibrillation capabilities could be an effective and safe therapeutic option. Unlike external electrical cardioversion, this strategy does not interfere with the implantable cardioverter defibrillator, is more effective, and obviates the need of general anesthesia. This strategy should be further evaluated in clinical trials.

## Case report

A 73-year-old Caucasian man presented to the outpatient clinic for evaluation of a recent episode of implantable cardioverter defibrillator (ICD) shock therapy. He had been implanted a dual chamber ICD (Model 1871, Vitality DR, Guidant Corp.) for secondary prevention due to resuscitated sustained ventricular tachycardia, not related to a correctable cause, in the context of severe ischemic cardiomyopathy. His past medical history was significant for coronary artery disease (old myocardial infarction and coronary artery bypass surgery), hypertension, diabetes mellitus, and hyperlipidemia. The patient's medications included metoprolol, ramipril, glimepiride, aspirin, and simvastatin.

**Figure 1:**
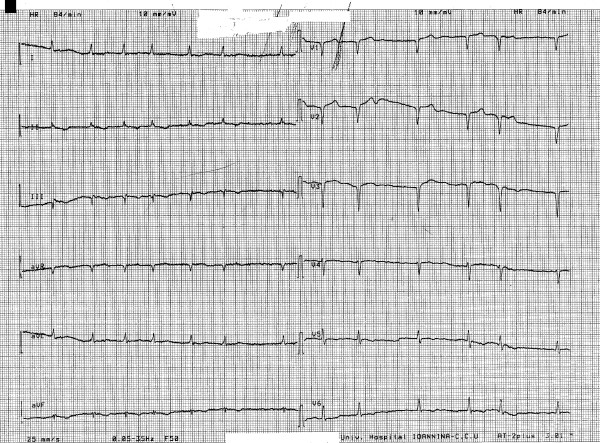
12-lead electrocardiogram of the patient at presentation.

**Figure 2:**
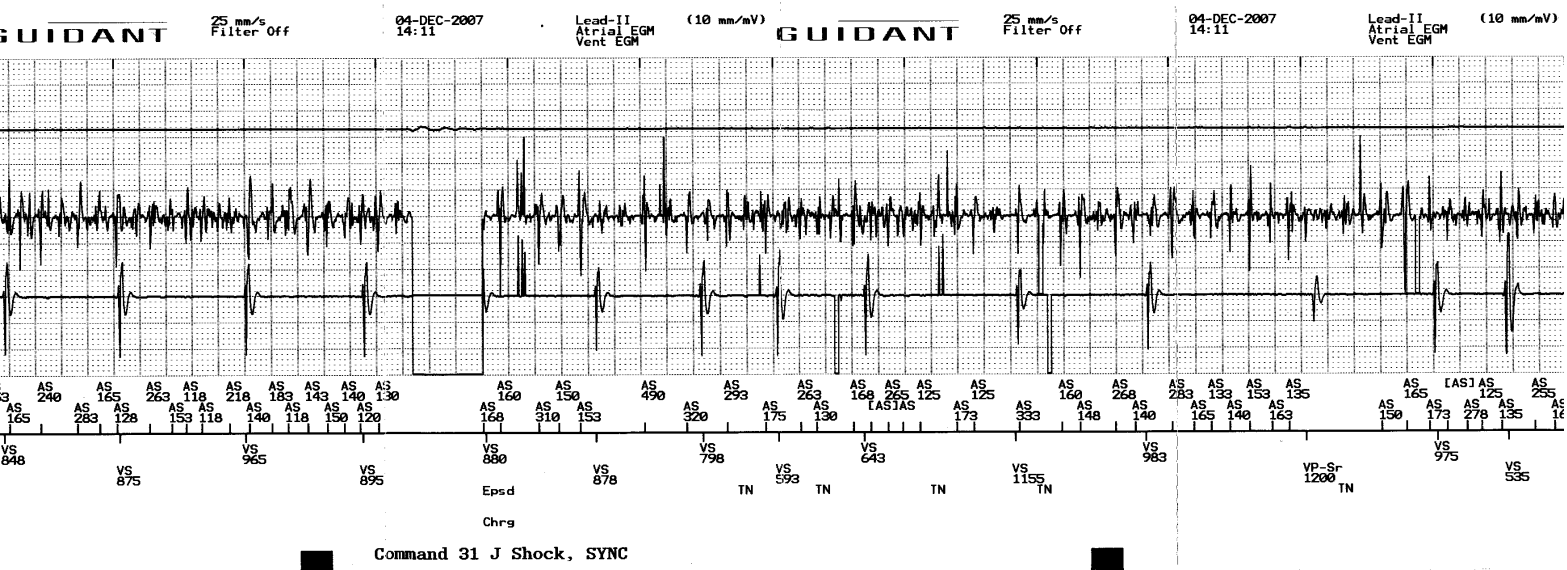
Interrogation of the ICD before cardioversion – Electrogram strip showing atrial fibrillation.

Interrogation of the stored events revealed that the recent episode of shock was an appropriate defibrillation (21 J biphasic shock) of ventricular arrhythmia (cycle length 330 ms) that classified into the VF zone. However, electrocardiographic examination and evaluation of the current electrograms revealed the presence of atrial fibrillation (AF) with a ventricular response of 84 beats/min (Figures 1, 2). The duration of AF was unknown while no event of inappropriate shock attributed to AF was detected. All hematological and biochemical studies including thyroid function tests were within normal limits. An echocardiographic study showed left ventricular (LV) dilatation with global systolic dysfunction (ejection fraction: 0.20) and evidence of increased filling pressures. The left atrial (LA) anteroposterior diameter was 41 mm and the LA diastolic volume 36 ml.

**Figure 3:**
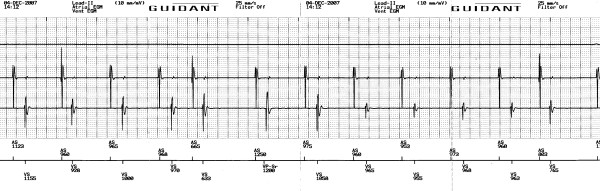
Electrogram strip after cardioversion of atrial fibrillation.

Taking into account the severely impaired left ventricular systolic function as well as the absence of LA enlargement we decided to follow a rhythm control strategy. Therefore, the patient was placed on appropriate anticoagulation therapy for 4 weeks and scheduled for cardioversion. He was admitted to the Coronary Care Unit and placed to electrocardiographic, non-invasive hemodynamic, and respiratory monitoring. After performing mild sedation with midazolam, the patient's ICD was externally programmed to deliver an R-wave synchronized ventricular biphasic shock of 31 J. The cardioversion was successful with immediate restoration of sinus rhythm (Figure 3). Subsequently, the patient was placed on amiodarone for sinus rhythm maintenance while continued receiving b-blocker therapy and anticoagulation. His recovery was rapid and uneventful and discharged 6 hours later. After a 10-month follow-up period, the patient remains on sinus rhythm and on good clinical condition. His LV ejection fraction has been improved to 0.28 while the LV filling pressures are normal. Interestingly, the patient does not remember any shock or associated pain with respect to the AF cardioversion.

## Discussion

ICD implantation rates are growing rapidly both for primary and secondary prevention of sudden cardiac death [1,2]. Given that ICD implantation is increasingly applied in patients with advanced structural heart disease, the prevalence of AF in ICD patients has been increased. Of note, a significant proportion of ICD patients have a history of AF or will develop AF after implantation while paroxysmal or persistent AF in ICD patients has been associated with subsequent appropriate therapy for ventricular arrhythmias [3-5]. Despite technological advances and sophisticated algorithms for improved discrimination between supraventricular and ventricular arrhythmias, inappropriate shocks represent a continuous problem in ICD patients and AF is the most frequent cause [6].

On the other hand, it is well-known that AF adversely affects myocardial performance, exercise tolerance, and quality of life, especially in patients with heart failure [7]. Moreover, AF per se is associated with increased morbidity and mortality mainly due to embolic events [7]. During the past few years ICDs that offer atrial defribrillator therapy have been developed. However, these devices have not gained wide acceptance [8]. The main reason for this fact is that the conscious patients experience pain during the atrial defibrillation shock. Even low energy shocks in the order of 1 J are uncomfortable for the patients leading to a significant impairment of the quality of life [8]. In other words, their shocks are considered as painful as the standard ICD ventricular defibrillations [8].

Synchronized electrical cardioversion is the preferable choice of therapy for persistent AF while the pharmacological cardioversion has limited role in this form of AF [9,10]. In specific, internal defibrillation shocks are preferable and more effective compared to external shocks in ICD patients [10]. Low energy delivered by the internal defibrillation lessens the risk for interference with the ICD programming as well as the risk for endocardial damage whereas the need for general anesthesia is obviated.

## Conclusion

Our case demonstrates that programmed ventricular ICD discharge under mild sedation could be an interesting option for quick, effective, and safe cardioversion of persistent AF. Taking into account the aforementioned considerations, this practice may have a particular role in patients with advanced heart failure. Undoubtedly, this strategy should be further evaluated in clinical trials.

## Consent

Written informed consent was obtained from the patient for publication of this case report and accompanying images. A copy of the written informed consent is available for review by the Editor-in-Chief of this journal.

## Competing interests

The authors declare that they have no competing interests.

## Authors' contributions

PK managed the patient, analyzed and interpreted the patient data, and he was a major contributor in writing the manuscript. GG managed the patient and involved in drafting the manuscript. TP involved in the care of the patient and in the drafting the manuscript. JAG searched the relative literature and critically revised the manuscript. All authors read and approved the final manuscript.
